# Supplementary material for: Determinants of depression among ever-married adolescent girls in Bangladesh: Evidence from the Bangladesh Adolescent Health and Wellbeing Survey 2019–2020
Source: PLoS One. 2024 Nov 25;19(11):e0314283. doi: 10.1371/journal.pone.0314283 (PMC11588215; doi:10.1371/journal.pone.0314283)
Supplement: S2 File — (PDF) [file pone.0314283.s002.pdf]

BANGLADESH ADOLESCENT HEALTH AND WELLBEING SURVEY (BAHWS) 2018-19

QUESTIONNAIRE FOR EVER MARRIED GIRL (15-19 YEARS)

TYPE TWO QUESTIONNAIRE

National Institute of Population Research and Training (NIPORT)
Medical Education and Family Welfare Division
Ministry of Health and Family Welfare

icddr,b

MEASURE Evaluation

Human Development Research Centre

FACE SHEET

IDENTIFICATION

CLUSTER NUMBER...	☐☐☐☐
HOUSEHOLD NUMBER..	☐☐☐
NAME OF HOUSEHOLD HEAD	
NAME AND LINE # OF RESPONDENT ______________________	☐☐

INTERVIEWER VISITS

	1	2	3	FINAL VISIT
DATE				DAY ☐☐<br>MONTH ☐☐<br>YEAR ☐☐☐☐
INTERVIEWER'S NAME				INT. CODE ☐☐☐
RESULT*				RESULT* ☐
NEXT VISIT: DATE<br>TIME	________<br>________	________<br>________		TOTAL NO. OF VISITS ☐

*RESULT CODES:
1 COMPLETED
2 NOT AT HOME
3 POSTPONED
4 REFUSED
5 PARTLY COMPLETED
6 INCAPACITATED
7 OTHERS______________________________
(SPECIFY)

SUPERVISOR	FIELD EDITOR	ICDDRB MONITORING OFFICER	OFFICE EDITOR	KEYED BY
NAME___________<br>☐☐☐<br>DATE___________	NAME___________<br>☐☐☐<br>DATE____________	NAME___________<br>☐☐☐<br>DATE___________	☐☐	☐☐

Consent of participant for individual information

Assalamualikum/Adab. My name is__________________________. The National Institute of Population Research and Training (NIPORT) of the Ministry of Health, Government of Bangladesh is conducting a national survey on adolescents' health and wellbeing. On behalf of NIPORT, I come from Human Development Research Centre (HDRC), a private research organization, to collect some information. The information will help to improve adolescent health and wellbeing in Bangladesh. We are conducting the survey in collaboration with icddr,b and the University of North Carolina, USA. You have been selected as respondents in this study.

The survey aims to understand the state of adolescent health and some aspects of wellbeing in Bangladesh.

If you agree to participate in this survey, I will ask some questions about your health and wellbeing. This questionnaire will take 40-45 minutes to complete. Participation in this survey is voluntary and you can choose not to answer any individual question or all the questions. However, we hope that you will participate in this survey since your views are important.

By providing information you will not have any risk whatsoever, rather this will help the government and policy planners to evaluate, strengthen and refocus national efforts for adolescent health and wellbeing.

Whatever information you provide will be kept strictly confidential. It will be used for research purposes. There are no financial benefits associated with your participation.

If you wish to know more about the survey, you can contact the following numbers:

NIPORT,: 0258611206 (Director , Research), 01552356842 (Evaluation Specialist)

icddr,b: 01777790154 (Research Investigator), 029886498/3206, 01711428989 (IRB Secretary).

Do you want to ask me anything about the interview/survey?

Do you agree to participate in this survey?

Yes = 1 ↓ No = 2 → END

Participant's Name: ___________________ Signature (or thumb print): ____________ Date: _______

Name of witness: _______________________ Signature: _______________ Date: __________

Name of person obtaining consent: _______________ Signature: ___________ Date: _______

Section 1: Respondent's Background

NO.	QUESTIONS AND FILTERS	CODING CATEGORIES	SKIP
101.	Record the time started	Hour [ ][ ] Min [ ][ ]	
102.	In what month and year were you born?	Month.. [ ][ ]<br><br>Don't Know Month...........................98<br><br>Year .... [ ][ ][ ][ ]<br><br>Don't Know Year.........................9998	
103.	How old were you at your last birthday? [What is your age?]<br><br>COMPARE AND CORRECT 102 AND /OR 103 IF INCONSISTENT	Age (In Completed Years) ………… [ ][ ]	
103a	Are you now married, separated, deserted, divorced, and widowed?	Currently Married………………...................1<br>Separated………………………...................2<br>Deserted………………………….................3<br>Divorced……………………….....................4<br>Widowed………………………......................5	2–5 → 104
103b	How old is your (current) husband?	Age (In Completed Years) ………… [ ][ ]	
104.	Have you ever attended a School, College or Madrasha?	Yes, School or College..............................1<br>Yes, Madrasha...2<br>Yes, School,College and Madrasha............3<br>No...4	1–2 → 106<br><br>4 → 109
105.	What type of school/college or Madarasa have you last attended?	School or College.............................1<br>Madrasha..2	
106.	What is the highest class you have completed at that School/ College or Madrasa?<br><br>IF COMPLETED LESS THAN ONE YEAR AT THAT LEVEL, RECORD '00'.	Class........................... [ ][ ]	
107.	Are you currently attending a School or College /Madrasha?	Yes, School or College..............................1<br>Yes, Madrasha...2<br>No...3	1–2 → 109
108.	For what reasons currently are you not attending School/ College or Madrasha?<br><br>MULTIPLE ANSWERS ACCEPTABLE	Distance to school.....................................A<br>Concern about safety.................................B<br>Parents concern: School quality..................C<br>Parents lack of interest...............................D<br>Poor performance in school.........................E<br>No good school in the locality......................F<br>Didn't know about school............................G<br>Student: Lack of interest.............................H<br>Got married...I<br>Had to care for siblings/ others....................J<br>Financial constraints/costs...........................K<br>Illness: family/respondent............................L<br>Household Chores/Work.............................M<br>Husband oppose..N<br>Have to take care of child............................O	

NO.	QUESTIONS AND FILTERS	CODING CATEGORIES	SKIP
		Other (specify)..X<br>Don't know..Z	
109.	Aside from housework, some persons take up jobs for which they are paid in cash or kind. Are you currently involved in any kind of work paid in cash or kind?	Yes, paid in cash.......................................1<br>Yes, paid in kind ……………………..………2<br>Yes, paid in both cash and kind……......……3<br>No..4	111
110.	Within the last six months, have you been involved in any kind of work for which you were paid in cash or kind?	Yes, paid in cash....................................1<br>Yes, paid in kind …………………………2<br>Yes, paid in both cash and kind…………3<br>No...4	112
111.	From where do you/did you do this work?<br>At home, outside or both?	At home..1<br>Outside home...2<br>Both...3	
**Now I want to talk about the family members that constitute the household you live in most of the time. Every household is different. Some adolescents get to live with both the parents in the same household, while others get to live with either one of them. I'd like to know a bit more about them and the other family members that make up your household.**			
112	Does your …… (name below) usually live in same household with you?		
a	Does your mother (biological mother) usually live in the same household with you?	YES...1<br>NO...2	c
b	You told that your mother doesn't live with you, Do you have step mother? If yes does your step mother usually live in the same household with you?	YES...1<br>NO...2<br>I don't have step mother..9	
c	Does your father (biological father) usually live in the same household with you?	YES...1<br>NO...2	e
d	You told that your father doesn't live with you, Do you have step father? If yes does your step father usually live in the same household with you?	YES...1<br>NO...2<br>I don't have step father...9	
e	Does your older brother usually live in the same household with you?	YES...1<br>NO...2	
f	Does your older sister usually live in the same household with you?	YES...1<br>NO...2	
g	Does your husband usually live in the same household with you?	YES...1<br>NO...2	
h	Does your mother-in-law usually live in the same household with you?	YES...1<br>NO...2	
i	Does your father-in-law usually live in the same household with you?	YES...1<br>NO...2	
**Now I am going to ask you about your engagement with any creative activities, cultural activities and/or outdoor sports. I also want to ask you about your affiliation with any club.**			

NO.	QUESTIONS AND FILTERS	CODING CATEGORIES	SKIP
125	**Are you currently engaged in any creative, cultural activities or outdoor sports such as**		
a	Drawing/painting	YES.......................................1<br>NO...2	
b	Singing /Dancing /Drama	YES.......................................1<br>NO...2	
c	Outdoor Sports such as football, cricket, bou-chi, hadudu/cabady, table tennis, badminton etc	YES.......................................1<br>NO...2	
d	Reading books other than textbooks	YES.......................................1<br>NO...2	
e	Creative Writing such as writing stories, poem etc	YES.......................................1<br>NO...2	
f	Other (Specify):	YES.......................................1<br>NO...2	
126	**Are you currently affiliated with any club or organization such as**		
a	Local Youth Club	YES.......................................1<br>NO...2	
b	Boys Scout/Girls Scout/ Girls Guide	YES.......................................1<br>NO...2	
c	Cultural Organization: (for example singing, dancing, drama, poetry, painting, drawing etc.)	YES.......................................1<br>NO...2	
d	Sports Club: school/college or community level	YES.......................................1<br>NO...2	
e	Reading Club/ Library	YES.......................................1<br>NO...2	
f	Other (Specify):	YES.......................................1<br>NO...2	
**Nowadays, many organizations (such as Government, NGO, and School) are running adolescent programs where they teach about adolescent rights, nutrition, vocational training, reproductive health such as puberty, menstruation, marriage, family planning and other life skills. Now, I would want to know if you have ever been involved in any such adolescent program currently or previously.**			
127	Are you currently involved with any adolescent program?	YES.......................................1 →<br>NO...2	201
128	In the last 3 years were you involved in any adolescent program?	YES.......................................1<br>NO...2	

Section 2: Exposure to Media

NO	QUESTIONS AND FILTERS		CODING CATEGORIES	SKIP
**Now I would like to ask you few questions about your exposure to Mobile phone, Internet, Radio, Television and newspaper.**				
201.	Do you have your own mobile phone that is currently functional?<br><br>[If YES, then ASK; is it a basic mobile phone or a smart phone?		Yes, basic mobile phone......................1<br>Yes, smart phone................................2<br>Yes, both basic and smart phone..........3<br>No...4	1-3 → 204
202.	Do any of your family members currently own a functional mobile phone?		YES..1<br>NO..2	2 → 206
203.	Do you have access to a family member's mobile phone in case you want to?		YES..1<br>NO..2	2 → 206
**Mobile phones are used to make and receive phone calls, text messages, access the internet etc. Now I want to know if you use the mobile phone for certain purposes.**				
204.	**Do you use the mobile phone to…..**		**205 If yes, do you use this every day or at least once a week or less than once a week**	
a.	Do you use the mobile phone to make and/or receive phone calls?	Yes……….1<br>No………..2 ↓	Everyday………………………………1<br>At least once a week…………………2<br>Less than once a week………………3	
b.	Do you use the mobile phone to send and/or receive texts?	Yes……….1<br>No………..2 ↓	Everyday………………………………1<br>At least once a week…………………2<br>Less than once a week………………3	
c.	Do you use the mobile phone to listen radio/?	Yes……….1<br>No………..2 ↓	Everyday………………………………1<br>At least once a week…………………2<br>Less than once a week………………3	
d.	Do you use the mobile phone to access the internet/Google/Youtube?	Yes……….1<br>No………..2 ↓	Everyday………………………………1<br>At least once a week…………………2<br>Less than once a week………………3	
e.	Do you use the mobile phone to read newspaper/magazine?	Yes……….1<br>No………..2 ↓	Everyday………………………………1<br>At least once a week…………………2<br>Less than once a week………………3	
f.	Do you use the mobile phone to use Facebook?	Yes……….1<br>No………..2 ↓	Everyday………………………………1<br>At least once a week…………………2<br>Less than once a week………………3	
g.	Do you use the mobile phone to use IMO/Viber/WhatsApp/Messenger?	Yes……….1<br>No………..2 ↓	Everyday………………………………1<br>At least once a week…………………2<br>Less than once a week………………3	
All this time I listened to your use of mobile phone. Now I want to know about your use of Laptop/Computer or Tab (Tablet)				
206.	Do you use internet through a Laptop/computer/tablet either in the house or elsewhere?<br><br>How often do you use internet through a computer/tablet: everyday, at least once a week, less than once a week or not at all?		Everyday..1<br>At least once a week……………………2<br>Less than once a week…………………3<br>Not at all…………………………………4	
207.	In the last 3 months, have you read any adolescent focused article online/internet, through mobile phone, laptop/ computer or tab?		YES...1<br>NO..2	

208.	Do you listen to the radio?<br><br>How often do you listen to the radio: everyday, at least once a week, less than once a week or not at all?	Everyday..1<br>At least once a week ....2<br>Less than once a week......................3<br>Not at all ....4 →	210
209.	In the last 3 months, did you listen to any adolescent focused program on the radio?	YES...1<br>NO...2	
210.	Do you watch television?<br><br>How often do you watch television: everyday, at least once a week, less than once a week or not at all?	Everyday..1<br>At least once a week ....2<br>Less than once a week......................3<br>Not at all ....4 →	212
211.	In the last 3 months, did you watch any adolescent focused program in TV?	YES...1<br>NO...2	
212.	Do you read printed version of newspaper/magazine?<br><br>How often do you read: everyday, at least once a week, less than once a week or not at all?	Everyday..1<br>At least once a week ....2<br>Less than once a week......................3<br>Not at all ....4 →	409
213.	In the last 3 months, have you read any adolescent focused news article or newspaper or magazine?	YES...1<br>NO...2	

Section 4: Marriage

NO.	QUESTIONS AND FILTERS	CODING CATEGORIES	SKIP
**For many people marriage is an important aspect of life. In the following section, I will ask you a few questions on marriage to gain a better understanding of your thoughts about this.**			
409.	**Check 103a:**<br>Currently Married ☐ ↓	Separated/ Deserted/ Divorced/ Widowed ☐ →	507
410.	Is your husband living with you now or is he staying elsewhere?	Living with her……………………………….1 →<br>Staying elsewhere, within Bangladesh…….2<br>Staying elsewhere, outside Bangladesh…..3	413
411.	How many times did he come, or you met with him in the past 12 months?	Number of times……………………… ☐☐<br>Did not come/met in the last 12 months…..00	
Now I will ask you some question about your marriage. In some cases, people get married more than once, and no one to be blamed for this or nothing should be ashamed of this. Please don't be ashamed.			
413.	Have you been married only once or more than once?	Only once . . . . 1<br>More than once 2	
414.	**Check 413:**<br>Married only once ☐ ↓ Ask: In what month and year did you get married?<br>Married more than once ☐ ↓ In what month and year were you married to your 1st husband?	Month... ☐☐<br>Don't know month....................................98<br>Year .... ☐☐☐☐<br>Don't know year......................................9998	
415.	How old were you when you (first) got married?	Age (In Completed Years) …………. ☐☐	
416.	Did you start living with your (first) husband the day you got married?	Yes…………………………………………….1 →<br>No……………………………………………….2<br>Did not start living with husband yet……….3 →	**418**<br><br>**418**
417.	After how many days/months/years of your marraige did you start living with your (first) husband?<br><br>***(If response is in month, convert to year and write)***	Months…… ☐☐ Days……… ☐☐	
418.	Do you think you got married at an age that was right for you or would you have preferred to marry earlier or later?	Right time..1 →<br>Earlier...2<br>Later...3	507
419.	At what age would you have prefered to get married?	Age (In Completed Years) …………. ☐☐	

Section 5: Reproductive history, Contraception and Fertility preference

NO	QUESTION AND FILTER	CODING CATEGORY	SKIP
**Now I would like to ask about all the births you have had during your life.**			
507.	If you have given birth in your lifetime I want to ask you more regarding those births.<br><br>Have you ever given birth?	Yes ....1<br>No..2 →	522
508.	Do you have any sons or daughters to whom you have given birth who are living with you?	Yes...1<br>No..2 →	510
509.	a) How many sons live with you?<br>b) And how many daughters live with you?	a) Sons living at home……. [ ][ ]<br>b) Daughters living at home [ ][ ]	
510.	Do you have any sons or daughters to whom you have given birth who are alive but do not live with you?	Yes...1<br>No..2 →	511a
511.	a)How many sons are alive but do not live with you?<br>b) And how many daughters are alive but do not live with you?	a) Sons live elsewhere……. [ ][ ]<br>b) Daughters live elsewhere [ ][ ]	
511a	How many boys and girls are alive?<br>"If she has no alive child; write 00"	Total number of living children [ ][ ]	
512.	Have you ever given birth to a boy or girl who was born alive but later died?<br><br>IF NO, PROBE: Any baby who cried, who made any movement, sound, or effort to breathe, or who showed any other signs of life even if for a very short time?	Yes...1<br>No...2 →	514
513.	a) How many boys have died?<br>b) And how many girls have died?	a) Boys died…………… [ ][ ]<br>b) Girls died…………… [ ][ ]	
514.	SUM ANSWER TO 509, 511 AND 513, AND ENTER TOTAL	Total Births………………. [ ][ ]	
515.	CHECK 514:<br>Just to make sure that I have this right: you have had in TOTAL______births during your life. Is that correct?<br><br>YES [ ] ↓ NO [ ] →<br>PROBE AND CORRECT 507-514 AS NECESSARY		

NO	QUESTION AND FILTER	CODING CATEGORY	SKIP

518	At what month and year your 1st child born?	Month ………………………… [ ][ ]<br>Month not known…………………98<br>Years ………………………… [ ][ ][ ][ ]<br>Year not known…………………9998	
519	How old were you when your 1st child born?	[ ][ ] Years<br>(Write in completed year)	
520	Did you prefer to have your 1st child at that age or you wanted to have earlier or later?	At that age……………………1<br>Wanted to have earlier……..2<br>Wanted to have later……….3	→ 522 (if 1)
521	When (at what age) would you have preferred to have your 1st child?	[ ][ ] Years<br>(Write in completed year)	
522	Are you currently pregnant?	Yes…………………………..1<br>No……………………………2<br>Unsure………………………8	
523	Check 507 and 522;<br>If 507=2 and 522=1 ↓<br>If 507=1 and 522=2 or 8 →<br>If 507=1 and 522=1 →<br>If 507=2 and 522=2 or 8 →		527<br>532<br>526
524	Did you want to get pregnant at this time?	Yes…………………………..1<br>No……………………………2	→ 532 (if 1)
525	When (at what age) would you have prefer to have your first child?	[ ][ ] Years<br>(Write in completed year)	→ 532
526	At what age do you want to take your first child?	[ ][ ] Years<br>(Write in completed year)	

NO	QUESTION AND FILTER	CODING CATEGORY	SKIP
**Now I would like to talk about family planning – The various ways or methods that a couple can use to delay or avoid a pregnancy**			
527.	Check Q103a<br>Currently Married ☐ ↓<br>Separated/Deserted/Divorced/Widowed ☐ →		710
528.	Are you or your partner currently doing something or using any method to delay or avoid getting pregnant?	Yes..1<br>No..2 →	531
529.	Which method are you using?<br><br>RECORD ALL MENTIONED	Female sterilization........................... A<br>Male sterilization............................... B<br>IUD .... C<br>Injectables .... D<br>Implants.. E<br>Pill..F<br>Condom.. G<br>Female condom.................................H<br>Emergency Contraceptive Pill (ECP)........I<br>Lactational Amenorrhea Method (LAM)....K<br>Rhythm Method..L<br>Withdrawal...M<br>(K, L, M → 536)<br><br>Other Modern Method (Specify) ....X<br>Other Traditional Method (Specify)..........Y	536
530.	Where did you obtain (Current method) the last time?<br><br>Probe to identify the type of source<br><br>***If unable to determine if the facility is a hospital, clinic, public, NGO or private, please write down the name of the facility below:***<br><br>……………………………………………<br>(Name of Facility).	**PUBLIC SECTOR**<br>Medical college/specialized hospital. 11<br>District hospital .... 12<br>Maternal child welfare center (MCWC)13<br>Upazila Health Complex....................14<br>Union Health & Family Welfare Centre / Rural dispensary/ Union Subcentre....15<br>Satellite clinic/EPI outreach site .... 17<br>Community clinic .... 18<br>Govt. field worker (FWA) .... 19<br>Other govt. (Specify)........................ 20<br><br>**NGO SECTOR**<br>NGO static clinic...............................21<br>NGO satellite clinic ....22<br>NGO depot holder ....23<br>NGO fieldworker...............................24<br>Other NGO (Specify)..............................26<br><br>**PRIVATE MEDICAL SECTOR**<br>Private hospital/clinic........................31<br>Qualified doctor's chamber...............32<br>Non-qualified doctor's chamber........33<br>Pharmacy/drug store ....34<br>Homeo/Herbal/Unani........................ 35<br>Other..36<br>(Specify)<br>**OTHER SOURCE**<br>Shop ....41<br>Friend/relatives ....42<br>Husband...43<br>Other..96<br>(Specify)	536

NO	QUESTION AND FILTER	CODING CATEGORY	SKIP
531.	Why are you and your husband not doing something or using any contraceptive method to delay or avoid getting pregnant?	General health concerns.......................A<br>Side effects..B<br>Difficulty in having sex..........................C<br>Interfered physiological normal processes...D<br>Did not like the method..........................E<br>Husband opposed.................................F<br>Others opposed....................................G<br>Social stigma..H<br>Religious prohibition...............................I<br>Currently pregnant..................................J<br>Want to have child now...........................K<br>Didn't find the appropriate FP method....L<br>Husband lives abroad.............................M<br>Other...X<br>(Specify)	
532.	Do you intent to use family planning method in the next 12 month?	Yes ....1<br>No...2<br>Haven't decided yet............................8	2, 8 → 534
533.	Which method do you prefer to use?<br><br>RECORD ALL MENTIONED	Female sterilization......................... ...A<br>Male sterilization................................ B<br>IUD .... C<br>Injectables .... D<br>Implants .... E<br>Pill...F<br>Condom .... G<br>Emergency Contraceptive Pill (ECP)...I<br>Lactational Amenorrhea Method(LAM)..K<br>Rhythm Method.....................................L<br>Withdrawal..M<br>Other Modern Method (Specify) ....X<br>Other Traditional Method (Specify)........Y	
534.	Have you ever used anything or tried in any way to delay or avoid pregnancy?	Yes ....1<br>No..2 →	537
535.	What method did you use?<br><br>RECORD ALL MENTIONED	Female sterilization.............................A<br>Male sterilization................................ B<br>IUD .... C<br>Injectables .... D<br>Implants .... E<br>Pill...F<br>Condom .... G<br>Emergency Contraceptive Pill (ECP)...I<br>Lactational Amenorrhea Method (LAM)...K<br>Rhythm Method......................................L<br>Withdrawal...M<br>Other Modern Method (Specify) ....X<br>Other Traditional Method (Specify).........Y	
536.	Would you say that using contraception is mainly your decision, mainly your husband's decision, or do you both decide together?	Mainly My Decision............................1<br>Mainly Husband.................................2<br>Both ....3<br>Other..6<br>(Specify)	

NO	QUESTION AND FILTER		CODING CATEGORY	SKIP
537	**Check 507 or 511 a**		None...00<br><br>Number.. ☐☐<br><br>Did not think of it yet...............................98	
	537 a.<br>Has a living child<br><br>511a=1 or more<br>☐	537b.<br>No living child<br><br>507=2 or 511a=0<br>☐		
	If you could go back to the time you did not have any children and could choose exactly the number of children to have in your whole life, how many would that be?	If could choose exactly the number of children to have in your whole life, how many would that be?		

Section 7: Connectedness: family and friends

NO.	QUESTIONS AND FILTERS	CODING CATEGORIES				SKIP
707	**PLEASE CHECK Q 103A**<br><br>**(Currently married)** ☐ ↓	**Separated/Deserted/Divorced/Widowed** ☐				**→710**
708	**Now I am going to read out some statements about your relationship with your husband and want to know which statement is applicable about you?**	**Never**	**Sometimes**	**Most of the times**	**Always**	
a.	You enjoy spending time with your husband	1	2	3	4	
b.	You and your husband are pretty close.	1	2	3	4	
c.	You talk with your husband about very personal things	1	2	3	4	
709	**Now I am going to mention some issues and ask you if you feel comfortable discussing those with your husband**	**Yes**		**No**		
a.	Menstruation	1		2		
b.	Progress in studies	1		2		
c.	When you want to have a child	1		2		
d.	Using family planning methods	1		2		
e.	Seeking health services for yourself	1		2		
f.	Participation in recreational activities: singing, dancing, drama, recitation of poem, drawing etc.	1		2		
g.	Participation in income generating activities outside home	1		2		
h.	Your Marriage	1		2		
i.	Intimate/Personal relationship	1		2		
j.	Sexual harassment : Sometimes we go through uncomfortable experiences inside and outside of home ; passing of sexual comments, sly whistle, physical touch that makes you uncomfortable, showing obscene photos, flashing of private parts, harassment through mobile call or text message.	1		2		
710	**CHECK 112a**<br>**Mother lives in the same household**<br>**Yes** ☐ ↓ **No** ☐ →<br>**Ask the following questions by referring to biological mother**	**CHECK 112h**<br>**mother in law lives in the same household**<br>**Yes** ☐ ↓ **No** ☐ →<br>**Ask the following questions by referring to Mother in law**				713

NO.	QUESTIONS AND FILTERS	CODING CATEGORIES	SKIP

NO.	QUESTIONS AND FILTERS	CODING CATEGORIES				SKIP
710.1	**Now I am going to read out some statements about your relationship with your mother/mother in law and want to know which statement is applicable about you?**	**Never**	**Sometimes**	**Most of the times**	**Always**	
a	You enjoy spending time with your mother/mother in law.	1	2	3	4	
b	You and your mother/mother in law are pretty close.	1	2	3	4	
c	You talk with your mother/mother in law about very personal things .	1	2	3	4	
711	**Now I am going to mention some issues and ask you if you feel comfortable discussing those with your mother/mother in law**	**Yes**		**No**		
a.	Menstruation	1		2		
b.	Progress in studies	1		2		
c.	When you want to have a child	1		2		
d.	Using family planning methods	1		2		
e.	Seeking health services for yourself	1		2		
f.	Participation in recreational activities: singing, dancing, drama, recitation of poem, drawing etc.	1		2		
g.	Participation in income generating activities outside home	1		2		
h.	Your Marriage	1		2		
i	Intimate/Personal relationship	1		2		
j	Sexual harassment : Sometimes we go through uncomfortable experiences inside and outside of home ; passing of sexual comments, sly whistle, physical touch that makes you uncomfortable, showing obscene photos, flashing of private parts, harassment through mobile call or text message.	1		2		
713	**CHECK: 112e and 123**<br>**If 112 e=1 or 123=1** ☐ ↓			**If 112e=2 and 123=2** ☐ →		**716**
714	**Now I am going to read out some statements about your relationship with your elder brother and want to know which statement is applicable about you?**<br><br>**(Instruction for interviewer : If respondent is not in touch with elder brother, left as a child and now not in touch, then code 'Not in touch with elder brother')**	**Never**	**Sometimes**	**Most of the time**	**Always**	**Not in touch with elder brother**

NO.	QUESTIONS AND FILTERS	CODING CATEGORIES				SKIP
a	You enjoy spending time with your elder brother	1	2	3	4	9 → 716
b	You and your elder brother are pretty close.	1	2	3	4	9
c	You talk with your elder brother about very personal things	1	2	3	4	9
715	**Do you feel comfortable discussing following issues with your elder brother?**	Yes		No		
a	Menstruation	1		2		
b	Progress in studies	1		2		
c	When you want to have a child	1		2		
d	Using family planning methods	1		2		
e	Seeking health services for yourself	1		2		
f	Participation in recreational activities: singing, dancing, drama, recitation of poem, drawing etc.	1		2		
g	Participation in income generating activities outside home	1		2		
h	Your Marriage	1		2		
i	Intimate/Personal relationship	1		2		
j	Sexual harassment : Sometimes we go through uncomfortable experiences inside and outside of home ; passing of sexual comments, sly whistle, physical touch that makes you uncomfortable, showing obscene photos, flashing of private parts, harassment through mobile call or text message.	1		2		
716	**CHECK: 112f and 124.**<br>**If 112 f=1 or 124=1** ↓	**If 112f=2 and 124=2** ☐ →				**719**
717	**Now I am going to read out some statements about your relationship with your elder sister and want to know which statement is applicable about you?**<br><br>**(Instruction for interviewer : If respondent is not in touch with elder sister, left as a child and now not in touch, then code 'Not in touch with elder sister')**	**Never**	**Sometimes**	**Most of the time**	**Always**	**Not in touch with elder sister**
a	You enjoy spending time with your elder sister	1	2	3	4	9 → 719
b	You and your elder sister are pretty close.	1	2	3	4	9
c	You talk with your elder sister about very personal things	1	2	3	4	9

NO.	QUESTIONS AND FILTERS	CODING CATEGORIES				SKIP
718	**Do you feel comfortable discussing following issues with your elder sister?**	Yes		No		
a	Menstruation	1		2		
b	Progress in studies	1		2		
c	When you want to have a child	1		2		
d	Using family planning methods	1		2		
e	Seeking health services for yourself	1		2		
f	Participation in recreational activities: singing, dancing, drama, recitation of poem, drawing etc.	1		2		
g	Participation in income generating activities outside home	1		2		
h	Your Marriage	1		2		
i	Intimate/Personal relationship	1		2		
j	Sexual harassments : Sometimes we go through uncomfortable experiences inside and outside of home ; passing of sexual comments, sly whistle, physical touch that makes you uncomfortable, showing obscene photos, flashing of private parts, harassment through mobile call or text message.	1		2		
719.	**Now I am going to read out some statements about your relationship with your friends.**<br><br>**You may consider anyone as your friend as for example your neighbours, school mates, cousins, play mates, or any individuals you communicate with.**<br><br>**Please take your time to understand the statements I will read out. Decide if the statement is true for you always, most of the time, sometimes or never**	**Never**	**Sometimes**	**Most of the time**	**Always**	**Does not have friends**
a.	You have friends that you consider close and trust completely	1	2	3	4	9 → 801
b.	Spending time with friends is important to you.	1	2	3	4	9
c.	You and your friends talk openly with each other about personal things.	1	2	3	4	9

Section 8: Gender norms

No.	QUESTIONS	CODING CATEGORIES		
**There are several opinions about the role and duties of a girl/female or a boy/male in our families and society. Now I am going to read out some statements and would like to know whether you agree or disagree with them.**				
**801**	**Gender attitudes**	**Agree**	**Disagree**	**Don't Know**
**A.**	It is important that sons have more education than daughters.	**1**	**2**	**8**
**B.**	Outdoor games are only for boys not for girls	**1**	**2**	**8**
**C.**	A family is not complete until they have at least one son.	**1**	**2**	**8**
**D.**	Household chores are for women only, not for men, even if the woman works outside the house.	**1**	**2**	**8**
**E.**	Women should not be allowed to work outside of home.	**1**	**2**	**8**
**F.**	A woman should always listen to her husband even if she disagrees.	**1**	**2**	**8**
**G.**	Looking after the household and kids is the responsibility of women only.	**1**	**2**	**8**
**H.**	Husband has the right to beat his wife when she does not obey him.	**1**	**2**	**8**

Section 9: Mental Health

<table>
<tr><th>NO.</th><th>QUESTIONS AND FILTERS</th><th colspan="4">CODING CATEGORIES</th></tr>
<tr><td colspan="6">Now I am going to discuss some mental health conditions that a person may experience. I want to know whether you experienced any such conditions in the last 2 weeks; if you have encountered these conditions, I would like to know how frequently you have experienced them in the last two weeks.

Instruction for interviewer: Please explain to the respondent what is meant by 2 weeks.</td></tr>
<tr><td>901</td><td>In the last two weeks till today how often have you.....</td><td>Not at all</td><td>Some days</td><td>Majority of the days</td><td>Nearly Everyday</td></tr>
<tr><td>a.</td><td>In the last two weeks till today how often have you felt down, depressed, irritable, or hopeless- Not at all, somedays, majority of the days or nearly every day?</td><td>0</td><td>1</td><td>2</td><td>3</td></tr>
<tr><td>b.</td><td>In the last two weeks till today how often have you had little interest or pleasure in doing things- Not at all, somedays, majority of the days or nearly every day?</td><td>0</td><td>1</td><td>2</td><td>3</td></tr>
<tr><td>c.</td><td>In the last two weeks till today how often have you had trouble falling asleep, staying asleep, or sleeping too much- Not at all, somedays, majority of the days or nearly every day?</td><td>0</td><td>1</td><td>2</td><td>3</td></tr>
<tr><td>d.</td><td>In the last two weeks till today how often have you had poor appetite, weight loss, or overeating- not at all, somedays, majority of the days or nearly every day?</td><td>0</td><td>1</td><td>2</td><td>3</td></tr>
<tr><td>e.</td><td>In the last two weeks till today how often have you felt tired, or had little energy- not at all, somedays, majority of the days or nearly every day?</td><td>0</td><td>1</td><td>2</td><td>3</td></tr>
<tr><td>f.</td><td>In the last two weeks till today how often have you felt bad about yourself – or felt that you are a failure- not at all, some days, majority of the days or nearly every day?</td><td>0</td><td>1</td><td>2</td><td>3</td></tr>
<tr><td>g.</td><td>In the last two weeks till today how often have you had trouble concentrating on usual activities- not at all, some days, majority of the days or nearly every day?</td><td>0</td><td>1</td><td>2</td><td>3</td></tr>
<tr><td>h.</td><td>In the last two weeks till today how often have you felt as if you have become more silent or restless- not at all, some days, majority of the days or nearly every day?</td><td>0</td><td>1</td><td>2</td><td>3</td></tr>
<tr><td>i.</td><td>In the last two weeks till today how often have you thoughts that you would be better off dead, or have hurt yourself in some way- not at all, some days, majority of the days or nearly every day?</td><td>0</td><td>1</td><td>2</td><td>3</td></tr>
<tr><td colspan="6">Instruction to Interviewer:

If 901i= 1,2 or 3, ask the respondent if he/she has discussed about this matter with anyone.
If the answer is “YES” (has discussed about this matter with someone) than advice him/her to continue the discussion with that person.
If the answer is “NO” then advice him/her to discuss the matter with someone he/she likes and trusts
Or call +880 ....</td></tr>
</table>

Section 10: Violence against adolescents, bullying and sexual harassment

1000	**Check Household Questionnaire Q20:**<br><br>**HH Q20=1** ☐ ↓ **HH Q20=2 or more** →	**1101**

READ TO RESPONDENT

Individuals including adolescents experience certain situations in their lives that are unpleasant and/or it make them uncomfortable. I would like to briefly ask about some of your own experiences and would like to know whether you have faced them in the last 12 months. I would also like to know from whom you have experienced it and how many times. You can talk to me openly about it and everything that you say will be kept confidential.

No.	QUESTIONS	CODING CATEGORIES	QUESTIONS	CODING CATEGORIES
1001	In the last **12 months**, has anyone acted in the following manner with you?		**1002**. In the past 12 months how many times did it happen?	**1003**. Who did this to you?<br>(Multiple answers acceptable)<br><br>Probe: Who else?
a	In the last **12 months**, has anyone slapped you, pushed you or pulled your hair?	Yes…1<br>No….2 ↓	Once………………...1<br>2-4 times…………….2<br>5 or more times……3	Father......................................A<br>Step Father………...................…B<br>Mother.....................................C<br>Step mother………...................…..D<br>Father in Law/ Mother in Law....E<br>Husband..................................F<br>Brother……………….…...........G<br>Sister.......................................H<br>Other Family Member/Relative..I<br>Teacher……………….…..…......J<br>Law Enforcement.………............K<br>Friends……………….….............L<br>School Peers……………..…..…..M<br>Neighbours…………….....….....N<br>Colleagues...............................O<br>Unknown person......................P<br>House holder/owner.................Q<br>Other (Specify)……........…......X
b	In the last **12 months**, has anyone punched, thrown something at you, hit you with a stick or something heavy?	Yes…1<br>No….2 ↓	Once………………...1<br>2-4 times…………....2<br>5 or more times……3	Father......................................A<br>Step Father………...................…B<br>Mother.....................................C<br>Step mother………...................…..D<br>Father in Law/ Mother in Law....E<br>Husband..................................F<br>Brother……………….…...........G<br>Sister.......................................H<br>Other Family Member/Relative...I<br>Teacher……………….…..…......J<br>Law Enforcement.………............K<br>Friends……………….….............L<br>School Peers……………..…..…..M<br>Neighbours…………….....….....N<br>Colleagues...............................O<br>Unknown person......................P<br>House holder/owner.................Q<br>Other (Specify)……........…......X

c	In the last **12 months**, has anyone kicked you, dragged you or, beat you up?	Yes…1<br>No….2 →	Once………………...1<br>2-4 times…………….2<br>5 or more times……3	Father......................................A<br>Step Father………..................…B<br>Mother.....................................C<br>Step mother…………....................D<br>Father in Law/ Mother in Law....E<br>Husband..................................F<br>Brother……………………..........G<br>Sister.......................................H<br>Other Family Member/Relative...I<br>Teacher……………….…...…....J<br>Law Enforcement……….............K<br>Friends……………………...........L<br>School Peers……………........…..M<br>Neighbours………….......….....N<br>Colleagues...............................O<br>Unknown person......................P<br>House holder/owner.................Q<br>Other (Specify)…….............…...X
d	In the last **12 months**, has anyone tried to choke you or burn you on purpose with something hot (fire, object, acid)	Yes…1<br>No….2 →	Once………………...1<br>2-4 times…………….2<br>5 or more times……3	Father......................................A<br>Step Father………..................…B<br>Mother.....................................C<br>Step mother…………....................D<br>Father in Law/ Mother in Law....E<br>Husband..................................F<br>Brother……………………..........G<br>Sister.......................................H<br>Other Family Member/Relative...I<br>Teacher……………….…...…....J<br>Law Enforcement……….............K<br>Friends……………………...........L<br>School Peers……………........…..M<br>Neighbours………….......….....N<br>Colleagues...............................O<br>Unknown person......................P<br>House holder/owner.................Q<br>Other (Specify)…….............…...X
e	In the last **12 months**, has anyone threatened or attacked you with a knife, gun or any other weapon	Yes…1<br>No….2 →	Once………………...1<br>2-4 times…………….2<br>5 or more times…….3	Father......................................A<br>Step Father………..................…B<br>Mother.....................................C<br>Step mother…………....................D<br>Father in Law/ Mother in Law....E<br>Husband..................................F<br>Brother……………………..........G<br>Sister.......................................H<br>Other Family Member/Relative...I<br>Teacher……………….…...…....J<br>Law Enforcement……….............K<br>Friends……………………...........L<br>School Peers……………........…..M<br>Neighbours………….......….....N<br>Colleagues...............................O<br>Unknown person......................P<br>House holder/owner.................Q<br>Other (Specify)…….............…...X

READ TO RESPONDENT

I have listened to the unwanted experiences that you might have faced in the last 12 months. Now, I would like to briefly ask you if anyone has cursed you, passed mean comments, called you names, had lies told about you, threatened you or excluded you socially in the last 12 months. You can talk to me freely about this and everything that you say will be kept confidential.

1004	In the last **12 months**, has anyone acted in the following manner with you?		1005. In the past 12 months how many times did it happen?	1006. Who did this to you? (Multiple answers acceptable)
a	In the last **12 months**, has anyone cursed you or passed mean comments or called you names?	Yes…1<br>No….2	Once………………...1<br>2-4 times…………….2<br>5 or more times……3	Father......................................A<br>Step Father………..................…B<br>Mother.....................................C<br>Step mother……......................…..D<br>Father in Law/ Mother in Law....E<br>Husband..................................F<br>Brother……………………............G<br>Sister.......................................H<br>Other Family Member/Relative...I<br>Teacher……………….....….......J<br>Law Enforcement………............K<br>Friends…………………..............L<br>School Peers………….........…..M<br>Neighbours…………….....…......N<br>Colleagues...............................O<br>Unknown person......................P<br>House holder/owner.................Q<br>Other (Specify)……........…......X
b	In the last **12 months**, has anyone blamed you to be liar or had lies told about you?	Yes…1<br>No….2	Once………………...1<br>2-4 times…………….2<br>5 or more times……3	Father......................................A<br>Step Father………..................…B<br>Mother.....................................C<br>Step mother……......................…..D<br>Father in Law/ Mother in Law....E<br>Husband..................................F<br>Brother……………………............G<br>Sister.......................................H<br>Other Family Member/Relative....I<br>Teacher……………….....….......J<br>Law Enforcement………............K<br>Friends…………………..............L<br>School Peers………….........…..M<br>Neighbours…………….....…......N<br>Colleagues...............................O<br>Unknown person......................P<br>House holder/owner.................Q<br>Other (Specify)……........…......X
c	In the last **12 months**, has anyone given you a written or verbal threat?	Yes…1<br>No….2	Once………………...1<br>2-4 times…………....2<br>5 or more times……3	Father......................................A<br>Step Father………..................…B<br>Mother.....................................C<br>Step mother……......................…..D<br>Father in Law/ Mother in Law....E<br>Husband..................................F<br>Brother……………………............G<br>Sister.......................................H<br>Other Family Member/Relative...I<br>Teacher……………….....….......J<br>Law Enforcement………............K

				Friends……………………...........L<br>School Peers…………......……..M<br>Neighbours……………...…......N<br>Colleagues...............................O<br>Unknown person......................P<br>House holder/owner.................Q<br>Other (Specify)……........…......X
d	In the last **12 months**, has anyone excluded you socially like did not include you while playing, did not let you sit with them or did not include you in idle chats.	Yes…1<br>No….2	Once………………...1<br>2-4 times…………....2<br>5 or more times……3	Other Family Member/Relative..A<br>Friends……………………...........B<br>School Peers…………......……..C<br>Neighbours……………...…......D<br>Colleagues...............................E<br>Unknown person......................F<br>House holder/owner.................G<br>Other (Specify)……........…......X
**READ TO RESPONDENT**<br>**I would like to briefly ask you if anyone has insulted you, made fun of you or spread false rumors about you using mobile SMS, MMS or Internet. You can talk to me freely about this and everything that you say will be kept confidential.**				
1007.	In the last **12 months**, has anyone acted in the following manner with you?		1008. In the past 12 months how many times did it happen?	1009. Who did this to you? (Multiple answer acceptable)
	In the last 12 months, has anyone used a mobile phone or internet to bother or harass you or to spread mean words or pictures about you in the last 12 months?	Yes…1<br>No….2	Once………………...1<br>2-4 times…………...2<br>5 or more times……3	Father......................................A<br>Step Father………....................…B<br>Mother.....................................C<br>Step mother…….....................…..D<br>Father in Law/ Mother in Law....E<br>Husband..................................F<br>Brother……………………..........G<br>Sister.......................................H<br>Other Family Member/Relative...I<br>Teacher…………………...……....J<br>Law Enforcement……….............K<br>Friends……………………...........L<br>School Peers…………......……..M<br>Neighbours……………...…......N<br>Colleagues...............................O<br>Unknown person......................P<br>House holder/owner.................Q<br>Other (Specify)……........…......X
**READ TO RESPONDENT:**<br><br>**A number of adolescents in their live have unwanted experiences by men/women and the culprit maybe be relatives, known or unknown persons. If you don't mind, I would like to briefly ask about some of these issues. Everything that you say will be kept confidential.**				

NO.	QUESTIONS AND FILTERS	CODING CATEGORIES	SKIP
**Now I'd like to ask you some questions about sexual harassment. Please do not feel shy about any questions, as the information you provide us would be very useful. Everything that you say will be kept confidential.**<br><br>**Instruction to interviewer**<br>**If all the response is "NO" skip to next section**			
**1010**	In the last **12 months**, has anyone acted in the following manner with you?		
**a.**	In the last **12 months**, has someone stared at you in a vulgar way that made you uncomfortable	YES..1<br>NO..2	
**b.**	In the last **12 months** have you encountered sly whistle/ humming of suggestive songs/or passing of sexual comments or jokes from someone	YES..1<br>NO..2	
**c.**	In the last **12 months** has someone touched you,/ grabbed you or pinched you in a way that made you uncomfortable	YES..1<br>NO..2	
**d.**	In the last **12 months** has someone forced you to watch obscene photos ,videos or flashed or mooned you?	YES..1<br>NO..2	
**e.**	In the last **12 months** did you face other similar experiences of sexual harassment?	YES..1<br>NO..2<br><br>If YES,<br>SPECIFY ___________________________	
**1011**	CHECK Q1010a to 1010e	Yes to any of the responses…………………………1<br>No to all responses………………………………..…2→	**1101**
**1012.**	(If any of the responses from 1010a to 1010e is yes)<br><br>Where did this harrasment take place?<br><br>CIRCLE ALL MENTIONED	At home...A<br>At School/College/Madrasha.................................B<br>On the road..C<br>Public Transport……………………………………..D<br>Marketplace…………………………………………..E<br>Neighbourhood……………………………...….…..F<br>Friends house………………………………………..G<br>Relatives house……………………………………...H<br>Coaching Centre……………………………………I<br>Workplace...J<br>Park or other other recreational place....................K<br>Health facility...L<br>Other(specify________________________)........X	

Section 11: Utilization of Health Services

NO.	QUESTIONS AND FILTERS	CODING CATEGORIES	SKIP
**Now I am going to ask you some questions regarding seeking information and/or service relating to health .**			
1101.	Did you visit any health facility in last 6 months for yourself?	Yes ....1<br>No……………………………………………..2	→ 1501
1102.	How many visits did you make?	___________ TIMES	
1103.	Where did you visit last?<br><br>***Probe to find out the correct health facility that was last visited and circle.***<br><br>***If unable to determine if the facility is a hospital, clinic, public, NGO or private, please write down the name of the facility below:***<br><br>……………………………………………<br>(Name of Facility).	**PUBLIC**<br>Public medical college/specialized hospital…….11<br>District hospital……………………………………12<br>Maternal child welfare center (MCWC)…………13<br>Upazila Health Complex…………………………14<br>Union Health & Family Welfare Centre/ Rural dispensary/ union sub centre……...............……15<br><br>School health clinic………………………………16<br>Satellite clinic/EPI outreach site…………………17<br>Community clinic…………………………………18<br>Other govt.______________………………………..20<br>(Specify)<br>**NGO**<br>NGO static clinic……………………………...……21<br>NGO satellite clinic..22<br>NGO depo holder..23<br>Other NGO (specify)______________...............26<br><br>**PRIVATE**<br>Private hospital/clinic/medical college…….……31<br>Qualified doctor’s chamber………………………32<br>Non-qualified doctor’s chamber…………………33<br>Pharmacy/drugstore………..……………………34<br>Homeopath/Herbal/Unani………………………..35<br>Other Private medical______________ ………..36<br>(Specify)	
1104.	In the last health facility you visited, is there a separate corner for adolescents to provide information and service?	Yes...1<br>No..2<br>Don’t Know..8	
1105.	In your last visit did you go for any information or services or both?	Information only...1<br>Service only...2<br>Both information and service................................3	

1106.	What information and/ or services did you receive the last time you visited?<br>Anything else?<br><br>***Do not read out responses.***<br><br><br>***Circle all mentioned.***	Menstrual problem management…………….....A<br>Anemia……………………………………………B<br>White discharge...C<br>Nutrition...D<br>Burning sensation during micturation………….E<br>General illness...F<br>Weakness..G<br>Allergy/Rash/Itching...H<br>Injury/Accident..I<br>Tetanus or Vaccination.…………………………..J<br>Diarrheal disease…………………………………K<br>Gastric problem……………………………………L<br>FP services………………………………………..M<br>Post abortion care………………..……………...N<br>Antenatal care…………………………………….O<br>Delivery……………………………………………P<br>Postnatal care……………………………………Q<br>Wet dream...R<br>Did not receive any treatment or service………S<br>Other_____________…………………………..X	

1501	End of interview	Hour ☐☐ Minute ☐☐
