# Supplementary material for: Determinants of depression among ever-married adolescent girls in Bangladesh: Evidence from the Bangladesh Adolescent Health and Wellbeing Survey 2019–2020
Source: PLoS One. 2024 Nov 25;19(11):e0314283. doi: 10.1371/journal.pone.0314283 (PMC11588215; doi:10.1371/journal.pone.0314283)
Supplement: Attachment — Submitted filename: Response to Reviewers.docx [file pone.0314283.s003.docx]

Dear Reviewers

Thanks for reviewing our manuscript titled “Determinants of Depression Among Ever Married Adolescent Girls in Bangladesh: Evidence from The Bangladesh Adolescent Health and Wellbeing Survey 2019-2020” and giving valuable comments. We put our best efforts to address all of your comments to meet the standard. We hope your thoughtful comments, queries and feedback have certainly enriched each and every section of our manuscript and made it more suitable for publication. Thanks once again for your valuable comments and observations which made our manuscript scientifically more improved.

As we mentioned earlier, we aim to investigate the determinants of depression among ever married adolescent girls in Bangladesh. It needs to be mentioned that there is a dearth of information regarding the prevalence and of determinants of depression among ever married adolescents in Bangladesh, utilizing national survey data. Therefore, this proposed paper is a unique to its own characteristics generating new knowledge by adding value to the scientific world.

Addressing all the comments, we are now resubmitting our revised manuscript. We hope that the revised manuscript has due merits to be published in journal. Please receive our revised manuscript along with necessary documents.

Please find below the responses against each of the observation and comment:

**Review of manuscript for PLOS ONE:** “Determinants of Depression Among Ever Married Adolescent Girls in Bangladesh: Evidence from Bangladesh Adolescent Health and Wellbeing Survey 2019-2020”

**Manuscript number:** PONE-S-24-05514

**# Journal requirements:**

**Comment:** Please ensure that your manuscript meets PLOS ONE's style requirements

**Response:** Thank you for your concern. We have taken a thorough look on the guideline and checked our formatting and styles in the manuscript as per journal requirements

**Comment:** Please provide a complete Data Availability Statement in the submission form, ensuring you include all necessary access information or a reason for why you are unable to make your data freely accessible. If your research concerns only data provided within your submission, please write "All data are in the manuscript and/or supporting information files" as your Data Availability Statement.

**Response:** Thank you for your suggestion. Since the data utilized in this study were extracted from a national survey (secondary source), therefore, we recommend restricting the data access to manuscript submission purposes only, rather than make it freely accessible. We put the recommended statement “All data are in the manuscript and/or supporting information files" in Data Availability Statement.

**# Reviewer(s)' Comments to Author:**

**Reviewer: 1**

**Overall**

**Comment:** It is a secondary data analysis. Then it should be described so.

**Response:** We appreciate your valuable feedback. We mentioned it in the introduction section
**Comment:** Introduction can be made more coherent.

**Response:** We appreciate your insightful feedback. We try our best to make this section more inclusive and coherent through comprehensive review
**Comment:** Check spelling (Syber Bullying)

**Response:** Many thanks, we corrected it accordingly
**Comment:** Inconsistent use of abbreviation (IPV not used even after prior elaboration earlier)

**Response:** We appreciate your insightful feedback. We checked this inconsistency and corrected accordingly
**Comment:** Statement in 346-347 needs reference

**Response:** Thanks for your insightful feedback. Line 346 indicates the association between sexual violence and depression that found in our study, therefore no reference is needed for this line, on the other hand, for line 347-348, we added the appropriate reference
**Comment:** Many mistakes in spelling-concrete 6.

**Response:** Thanks, we corrected the spelling accordingly
**Comment:** Meaning of Line 369-379 not clear

**Response:** Thanks for your excellent observation. Indeed, in our multivariate analysis (AOR- Table-3), education didn’t appear as significant factor in determining the depressive symptoms but appear significant in univariable analysis (COR- Table-3). Therefore, to support and justify our findings, we discussed the relevant literature on the association between education and depression in lines 369-389. In this part of discussion, we have edited few sentences to make it more precise and understandable.
**Comment:** Line 401 -should be though not through

**Response:** Thanks, we corrected the spelling accordingly.
**Comment:** Is it multivariate/univariate analysis? Use standard terms

**Response:** Thanks for your insightful feedback. In the statistical analyses section, we used the term 'univariable analysis' to represent the Crude Odds Ratio (COR), which does not account for any potential confounders in the model during the analysis (Table 3). On the other hand, we used 'multivariable analysis,' also referred to as 'multivariate analysis,' to indicate the Adjusted Odds Ratio (AOR) in the model, which accounts for potential covariates in the analysis (Table 3). Our findings in the manuscript were interpreted based on this multivariable/multivariate analysis.
**Comment:** The study is important, but the manuscript needs significant language edits and spell checks

**Response:** Thanks for your valuable feedback. We put our best efforts to navigate this problem and improve the flow. We have checked thoroughly for settling the grammatical and linguistic errors in our revised paper to make it easier for the readers.

**Reviewer: 2
Introduction
Comment:** The introduction is informative and thorough. However, streamlining certain sections could enhance readability. You might consider combining similar points and adopting more concise language to improve the flow.

**Response:** Thanks for your insightful comments. We have given a thorough look to fix all the inconsistencies including linguistics and grammatical mistakes throughout the introduction. Further, we have revised the flow of the introduction to make it easier to understand for the readers.
**Comment:** Introducing a theoretical framework could significantly bolster the study's conceptual foundation. For instance, applying the ecological systems theory or the life course perspective might provide valuable insights into the factors influencing depression among married adolescent girls.

**Response:** Thanks for your insightful suggestion. Yes, we follow a theoretical framework in the introduction section to have a solid foundation for conducting this study. The introduction section was systematically guided by a matrix/framework that are as follows: 1. describing the outcome of interest (depression) and its relevance; 2. describing the problem in global and national context as well as highlighted the relevance of the study by stating the available evidence and existing knowledge gap. 3. adopting a theoretical framework based on the socio-ecological model, which indicate individual’s mental health are influenced by multiple levels of indicators among the married adolescents in the context of Bangladesh, including individual, community, and societal (Socio-demographic) factors and discuss accordingly in the introduction section. For instance, we discuss the association between violence and different spectrums of mental health conditions among the married adolescents in Bangladesh. As well as we also discuss the social determinants of depression among the respondents in Bangladesh. 4. Discuss and highlight the significance of the current study by articulating the research gap among the married adolescents 5. Statement of the study objective.

**Comment:** To improve clarity and focus, explicitly stating the specific objectives of the study would be beneficial. While the introduction suggests the research aims, clear articulation of these objectives is recommended.

**Response:** Thanks for your insightful feedback. As recommended, we explicitly stated the specific objectives of the study
**Comment:** It would be helpful to include precise definitions of key terms such as "depression" and "adolescence." This will ensure consistency and clarity throughout the manuscript.

**Response:** Thanks for your insightful feedback. Yes, the elaborate and precise definition of these key terms was absent in the manuscript. As suggested, we elaborately defined the “depression, and “adolescence”, according to WHO definition
**Comment:** Enhance the connection between the identified risk factors for depression (e.g., social isolation, limited autonomy) and the specific context of married adolescent girls in Bangladesh.

**Response:** Thanks for valuable suggestion. As suggested, we discussed the relevant associations in the introduction section
**Comment:** Address potential ethical considerations pertinent to conducting research with this vulnerable population.

**Response:** Thanks for your concern. We conducted this analysis utilizing the national survey data (secondary source) for this study. The original cross-sectional survey obtained ethical approval from the relevant institutional review board and ensured that all respondents or their caregivers provided informed consent before participating in the interviews including the assessment of depression. The details of the ethical considerations, including the participant consent form, were submitted separately during the first submission. However, leveraging this secondary data in our analysis complies with the ethical standards for research involving human subjects, and no additional ethical approval was required for this secondary analysis.

**Methods**

**Comment:** Considering the complex sampling design, it would be helpful to indicate whether sample weights were applied in the analysis to account for the sampling design and enhance the representativeness of the results

**Response:** Thanks for valuable suggestion. Since our study utilized secondary data, we incorporated sampling weights in the regression model (Table-3) to enhance the robustness and generalizability of our findings. As suggested, we have included this information in the 'Statistical Analysis' section of the manuscript

**Comment:** The exclusion of 383 pregnant women and 394 cases with missing data is mentioned. Providing a more detailed explanation of the missing data mechanisms (e.g., missing completely at random, missing at random, missing not at random) would add clarity.

**Response:** Thanks for valuable suggestion. In the methodology section, we explicitly addressed the mechanisms of missing data. For instance, a total of 383 observations from pregnant women were excluded purposively due to the known association between depression and pregnancy/early childbearing. Additionally, 394 observations were excluded from the analysis due to missing information on harassment and violence indicators. Given that this missing data was random and unrelated to the outcome of depression, we applied a listwise deletion process to systematically exclude these observations to maintain the integrity of our results.

**Comment:** The information provided on the reliability and validity of the PHQ-9 is not adequate.

**Response:** Thanks for valuable suggestion. As suggested, we tried to provide detail information on PHQ-9.

**Comment:** Further elaboration on the rationale for selecting the specific covariates would be appreciated. Was the selection guided by a theoretical framework

**Response:** Thanks for valuable recommendations. We were guided by relevant literature from LMICs, that report the determinants of depression among adolescents. Based on this review, we considered the potential covariates for our study. Additionally, we employed a conceptual framework to select these covariates, focusing specifically on socio-demographic factors and indicators of violence, to select the relevant variables.

**Comment:** Clarifying how missing data for covariates were managed (e.g., imputation, listwise deletion) would strengthen the methodology section.

**Response:** Thanks for valuable suggestion. Due to 394 missing data points for physical and sexual harassment and other covariates, a listwise deletion process was applied. This approach systematically excluded 394 observations related to depression status to ensure the accuracy and completeness of the analysis.

**Discussion**

**Comment:** While the discussion provides a good overview of the associations between depression and the studied factors, it could be strengthened by exploring potential mechanisms underlying these relationships. For example, how might physical violence lead to depression?

**Response:** Thanks for your suggestion. Based on available literature, we explicitly discussed the potential pathways for the associations that induce depression among adolescents. Since, there is scarce of information in the context of Bangladesh regarding these pathways, therefore we recommend further research to explore the underlying mechanisms that lead to depression.

**Comment:** The discussion could be expanded to provide more specific policy recommendations based on the study findings. What types of interventions could be implemented to address the high prevalence of depression among married adolescent girls?

**Response:** Thanks for insightful recommendation We recommended implementing community-based screening programs for the early detection of depression as part of a primary prevention approach. Additionally, we suggest engaging community healthcare providers to ensure continuity of care reduce this the burden as part of secondary prevention approach. Moreover, we added a section namely “Policy implications” for elucidating these recommendations.

**Comment:** The authors could elaborate on the potential directions for future research to build upon the findings of this study. For example, longitudinal studies could be conducted to examine the causal relationships between the studied factors and depression.

**Response:** Thanks. We elaborately discussed this issue in the limitation section. Since we utilized survey/cross-sectional data for this study, that could not establish a causal or dose-response relationship between the risk factors and the outcome of interest (depression). Therefore, we recommend conducting longitudinal or follow-up studies to establish causal associations. Furthermore, we also recommend to explore the potential mediator/pathway of the determinants that later on lead to depression, to fill the knowledge gap and take appropriate interventions.

**Comment:** Consider incorporating a theoretical framework to guide the interpretation of the findings and to explain the potential mechanisms underlying the observed associations.

**Response:** We guided by a framework to interpret our findings align with each of the factors that led depression. For example, we explain how various factors—such as socio-demographic characteristics, exposure to violence, and harassment—affect mental health outcomes by considering the context, social norms, and practices in Bangladesh. By integrating this framework, we provided a comprehensive explanation of the pathways that lead to depression, supported by the associations observed in our study. We also discuss the strength and limitations of our observed findings and provide specific recommendations in terms of different prevention approaches.

**Comment:** Discuss the potential role of cultural factors in shaping the mental health experiences of married adolescent girls in Bangladesh.

**Response:** We discuss the different dimensions of cultural factors such as such as early marriage, IPV, inequality, poor subjective happiness and limited mobility that shaping and influencing the mental health status in the context of Bangladesh.

**Comment:** Provide more specific recommendations for interventions targeting different levels of prevention (primary, secondary, and tertiary) and for different stakeholders (e.g., government, healthcare providers, community organizations).

**Response:** We provided recommendations for preventing and controlling depression, focusing on various prevention mechanisms and engaging with different stakeholders. In addition, we added a section namely “Policy implications” for elucidating these recommendations.

**Conclusion**

**Comment:** While the conclusion calls for policy interventions, it could be strengthened by providing more specific recommendations. For example, the authors could outline potential policy targets, such as strengthening law enforcement against violence, increasing access to mental health services, or promoting gender equality.

**Response:** As recommended, we incorporated this into the conclusion. For example, we recommended introducing the screening program into the community level; enforce the strict law; and behavioral change interventions to prevent any form of violence and harassment

**Comment:** The conclusion could end with a strong call to action, emphasizing the urgency of addressing the mental health needs of married adolescent girls

**Response:** This issue is also mentioned in the section as suggested.

**FORMATTING AMENDMENTS (if any)**

1. We added “Policy Implication” in the discussion section as recommended by reviewer
